# The Influence of Carotid and Vertebral Doppler Ultrasonography and Brain MRI Abnormalities on Hearing Levels, Tinnitus Intensities and Frequencies

**DOI:** 10.3390/audiolres15020029

**Published:** 2025-03-15

**Authors:** András Molnár, Viktória Molnár, Panayiota Mavrogeni, Stefani Maihoub

**Affiliations:** 1 Protone Audio Kft., Opera Clinic, Lázár u. 4, H-1065 Budapest, Hungary; 2 Department of Otorhinolaryngology and Head and Neck Surgery, Semmelweis University, Szigony u. 36, H-1083 Budapest, Hungary; 3 Tóth Ilona Health Service, Clinical Medical Institute, Görgey Artúr tér 8, H-1212 Budapest, Hungary; 4 Maihoub ENT Clinic, Aliakmona Street 16, Cy-3117 Limassol, Cyprus; stephaniemaihoub@gmail.com

**Keywords:** tinnitus, pure-tone averages, tinnitus intensity, tinnitus frequency, carotid–vertebral Doppler ultrasonography, brain MRI

## Abstract

**Objectives**: This study aimed to analyse the potential influence of abnormalities detected through carotid–vertebral ultrasonography and brain MRI on pure-tone averages (PTAs) and the frequency and intensity of tinnitus. **Methods**: 423 participants with subjective tinnitus were enrolled in this investigation. All patients underwent carotid– vertebral ultrasonography, brain MRI, and pure-tone audiometry, including tinnitus matching. **Results**: The median values for tinnitus onset indicated chronic tinnitus in most cases. Regarding tinnitus location, left-sided symptoms (32%) and bilateral symptoms (44%) were the most prevalent. In analysing the effects of abnormalities detected by carotid–vertebral ultrasonography on PTAs, a statistically significant difference was found between the groups (*p* = 0.0037). Specifically, individuals with intimal hyperplasia had significantly higher PTAs (*p* = 0.02), as did those with carotid artery plaques (*p* = 0.005). However, no significant differences in PTAs were noted in relation to carotid artery stenosis (*p* = 0.07). Similar trends emerged regarding tinnitus intensity (*p* = 0.013), with significantly higher values observed in the presence of any carotid–vertebral ultrasonography abnormalities. In contrast, tinnitus frequencies were not significantly affected (*p* = 0.401). Regarding brain MRI findings, Fazekas scores of 2 (*p* = 0.02) and 3 (*p* = 0.0052) significantly influenced PTAs. For tinnitus intensity, Fazekas scores of 2 (*p* = 0.0027) and 3 (*p* = 0.0005), and the presence of acoustic neuromas *(p* = 0.019), significantly impacted the intensity values. However, tinnitus frequencies were not significantly (*p* = 0.36) influenced by brain MRI abnormalities. **Conclusions**: The findings of this study show that carotid–vertebral ultrasonography and brain MRI abnormalities significantly influence PTAs and tinnitus intensities.

## 1. Introduction

Tinnitus is the experience of hearing sounds that are not caused by any external source [1]. Its prevalence is increasing worldwide [2]. Tinnitus significantly impacts the quality of life and leads to psychiatric symptoms [3]. In cases of secondary tinnitus, a specific cause can be identified (e.g., outer or middle ear infections, otosclerosis, Ménière’s disease, or acoustic neuroma, etc.). However, primary tinnitus cases are often idiopathic, meaning no specific cause can be determined [4]. This category includes cases associated with sensorineural hearing loss, which significantly contributes to the occurrence of tinnitus [5]. Therefore, conducting hearing tests and tinnitus assessments is crucial for effectively managing patients who experience this condition. The exact cause of sensorineural hearing loss is frequently unclear. Key factors may include infections, exposure to ototoxic substances, circulatory issues, autoimmune conditions, endocrine disorders, neurological disorders, noise-induced trauma, and ageing, among others [6]. In everyday clinical practice, a specific cause for most cases of sensorineural hearing loss often remains unidentified. Carotid and vertebral Doppler ultrasonography is an examination commonly utilised to assess tinnitus [7,8] and sensorineural hearing loss [9], helping to identify potential vascular causes. Research indicates that atherosclerosis in the carotid and vertebral arteries accounts for 20% of ischemic strokes. Additionally, tinnitus, hearing loss, and vertigo can be early, non-specific symptoms of significant arterial stenosis and ischaemic stroke. These initial symptoms can be attributed to the ischaemic effects of the inner ear arteries [10]. Ultrasound tests can detect thickening of the arterial walls, the presence of arterial plaques, and stenosis, which may or may not be significant. These tests can also measure the velocities of arterial blood flow. Brain MRI examinations are primarily used to identify issues in the internal auditory canals and the cerebellopontine angles. This includes the detection of benign tumours, such as acoustic neuromas (vestibular schwannomas) and meningiomas [11]. Additionally, MRI can reveal other abnormalities, including vascular lesions and neurovascular conflicts, with or without evidence of compression [12]. The recommendations for routine brain MRI in cases of tinnitus vary among different authors. Some guidelines suggest that an MRI is necessary for unilateral tinnitus accompanied by unexplained hearing loss [13], while others recommend it for unilateral tinnitus regardless of whether there is hearing loss [14]. Although the abnormalities detected by brain MRI may often be incidental and not relevant, there are instances where significant changes can be observed, necessitating further evaluations [15]. Vascular lesions, often referred to as white matter lesions, are commonly observed, particularly with advancing age and vascular risk factors such as hypertension. However, these lesions have also been associated with impairments in neurological function [16]. Consequently, even incidental findings from an MRI can be clinically significant. Although various medical imaging techniques have identified abnormalities associated with tinnitus and sensorineural hearing loss, these findings have only been studied to a limited extent. Consequently, the relationship between these abnormalities and auditory symptoms is still unclear, including the effectiveness of treatments aimed at addressing these issues.

Given the high prevalence of abnormalities detected through carotid–vertebral Doppler ultrasonography and brain MRI, and their unclear effects on tinnitus and pure-tone averages (PTAs), this investigation aimed to analyse their correlations. The analysis focused on the potential effects of these abnormalities on PTAs, and on the intensity and frequency of tinnitus.

## 2. Material and Methods

### 2.1. Study Population

A total of 423 participants (median age: 51 years; 239 women) were enrolled in this study. The basic characteristics of the study population are presented in Table 1. Participants were evaluated for primary subjective non-pulsatile tinnitus, including cases linked to sensorineural hearing loss. Each participant underwent a detailed medical history assessment and a comprehensive otorhinolaryngological examination by a qualified specialist. Additionally, all participants underwent pure-tone audiometry testing and tinnitus evaluations, and carotid–vertebral Doppler ultrasonography and brain MRI, as described below. Every participant gave their written informed consent to participate in this study. Patients under 18 years of age and those who had undergone carotid endarterectomy, carotid angioplasty, stent placement, or any other surgeries were excluded from this investigation. The investigation followed the Declaration of Helsinki and received approval from the Hungarian ETT TUKEB (approval number: BM/29864–1/2024).

### 2.2. Medical Imaging

All participants underwent contrast-enhanced brain MRI examinations at 1.5 Tesla. These examinations included T2-weighted, FLAIR, SWI, and DWI imaging in the axial plane, as well as FLAIR images in the coronal plane. Prior to the contrast-enhanced exams, all patients had laboratory tests to ensure normal kidney function. The brain MRI results were evaluated by qualified neuroradiologists, focusing specifically on the internal auditory canals and cerebellopontine angle to identify any acoustic neuromas (vestibular schwannomas) or meningiomas in these areas. Microvascular lesions were categorised based on the Fazekas grading system, which organises them into stages 1, 2, or 3 [17]. Neurovascular conflicts involving the vestibulocochlear nerve and the anterior inferior cerebellar artery (AICA) were also assessed, with any potential compression ruled out. Additionally, carotid and vertebral ultrasonography was performed by a radiologist with the patient in a supine position. The procedure began with a transverse B-mode scan, followed by colour mode to identify blood flow. Measurements included the peak systolic and diastolic velocities of the common carotid, internal carotid, external carotid, and vertebral arteries on both sides. During the examination, structural changes were also assessed and categorised as intimal hyperplasia, plaque build-ups, or stenosis.

### 2.3. Pure-Tone Audiometry and Tinnitus Pitch-Matching

Before conducting pure-tone audiometry, the status of each patient’s external and middle ear was assessed, and tympanometry was performed to eliminate any potential causes of conductive hearing loss. Pure-tone audiometry was conducted using a GSI 61 Audiometer (Grason Stadler, Inc., Milford, CT, USA) in a soundproof booth. For each participant, a frequency range of 125 to 8000 Hz for air conduction and 250 to 4000 Hz for bone conduction, using increments of 5 dB, was analysed. This testing followed octave and inter-octave frequencies in accordance with the modified Hughson–Westlake procedure [18]. Air conduction testing was performed using headphones, while bone conduction was assessed with a mastoid vibrator. The right ear was always tested first. Averages from the pure-tone audiometry were calculated, following the recommendations of the Committee on Hearing and Equilibrium of the American Academy of Otolaryngology-Head and Neck Surgery, to clearly define sensorineural hearing loss [19]. Pure-tone audiometry was conducted by a qualified audiologist assistant, who manually scored each case.

Tinnitus pitch and intensity matching were conducted for the right ear, left ear, or both, depending on the laterality of the tinnitus. The pitch matching process covered a frequency range from 125 Hz to 8000 Hz and utilised the ‘bracketing’ method, starting at 1000 Hz. During the tinnitus pitch matching, participants listened to a sound stimulus and compared it to their perceived tinnitus, indicating whether the played sound was higher or lower in pitch. The frequency reported by the participants as matching their perception of tinnitus was recorded as the tinnitus frequency. Subsequently, the intensity of the tinnitus was measured in small increments of 1 dB. Both pitch and intensity matching procedures were performed three times using a forced-choice method. The results of the tinnitus matching process were documented on audiograms.

### 2.4. Statistical Analysis

All statistical analyses were conducted using IBM SPSS version 25 software (IBM Corporation, Armonk, NY, USA). The Shapiro–Wilk test was used to evaluate the data distribution, revealing that it was not normally distributed. Consequently, continuous variables were reported as medians with interquartile ranges (IQRs). To determine statistically significant differences, both the Shapiro–Wilk test and the Mann–Whitney *U* test were employed. Furthermore, Cohen’s *d* values were calculated to analyse effect sizes, with values of 0.2, 0.5, and 0.8 considered to represent small, medium, and large effect sizes, respectively. Furthermore, a multinomial logistic regression model has been utilised. Additionally, the relationships between the parameters were analysed using Spearman’s correlation test. The significance level was established at *p* < 0.05.

## 3. Results

Table 1 presents essential information about the current study population.

Table 1 indicates an increasing trend in the occurrence of tinnitus around the age of 50, with a notable predominance among females. When analysing the correlations between age and pure-tone audiometry results, significant relationships were found. Specifically, a correlation was found with PTAs (rho = 0.172, *p* = 0.001 *) and tinnitus intensities (rho = 0.477, *p* = 0.000 *), as determined by Spearman’s correlation test. However, tinnitus frequencies did not show a significant correlation with age (rho = −0.131, *p* = 0.106). The median values for tinnitus onset suggest that most cases are chronic. Regarding the location of tinnitus, the left side is the most commonly affected, accounting for approximately 32% of cases, while bilateral symptoms are observed in 44%. Pure-tone audiometry reveals that mild-to-moderate sensorineural hearing loss is the most prevalent category, with tinnitus intensities reported at similar levels. Additionally, tinnitus frequency tends to peak in the middle to high ranges.

**Figure 1:**
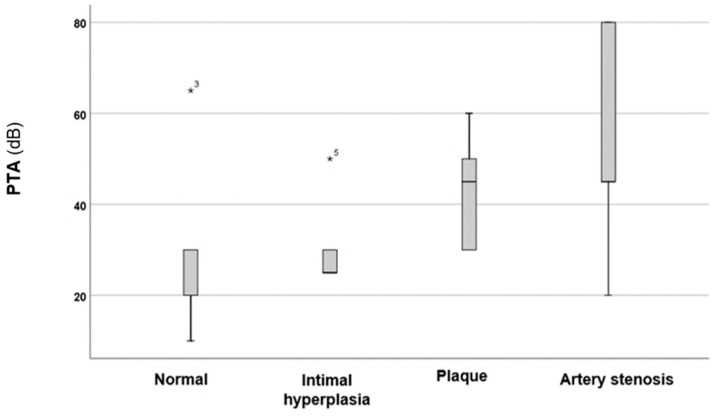
PTA values based on changes observed through carotid–vertebral Doppler ultrasonography. In the boxplots, the boxes represent the interquartile range of the data, while the whiskers indicate the lower and upper quartiles. The black line that divides the boxes marks the median values. Statistical differences were analysed using the Mann–Whitney *U* test and Kruskal–Wallis test (*p* < 0.05 *). dB = decibel; PTA = pure-tone average. The asterisks and numbers displayed in the figure denote the outliers.

**Figure 2:**
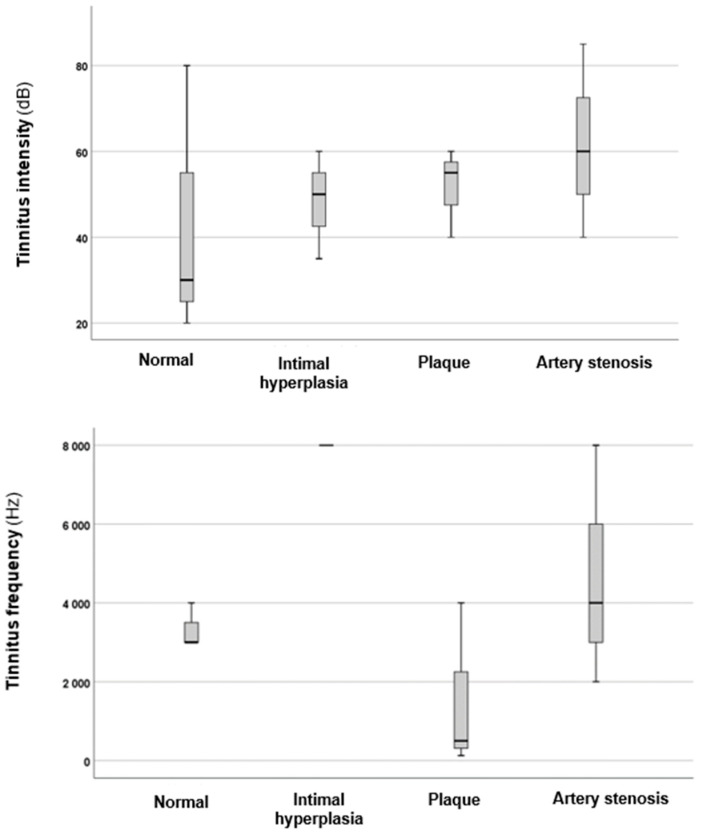
Tinnitus intensity and pitch values based on changes observed through carotid–vertebral Doppler ultrasonography. The black line that separates the boxes marks the median values. The statistical differences were analysed using the Mann–Whitney *U* test and Kruskal–Wallis test (*p* < 0.05). dB = decibel; Hz = Hertz.

In the next phase of the investigation, the effects of the carotid–vertebral ultrasonography results on PTAs, as well as the intensities and frequencies of tinnitus, were analysed. The results are illustrated in Figure 1 and Figure 2.

As illustrated in Figure 1, the severity of alterations observed in the carotid–vertebral ultrasonography was correlated with PTAs in patients. A statistical analysis using the Kruskal–Wallis test revealed a significant difference among the four groups (*p* = 0.0037 *; *H* = 13.46). Further analysis using the Mann–Whitney *U* test, which compared the group with normal results to the other groups, found statistically significant differences in intimal hyperplasia (*p* = 0.02 *, *z*-score: −2.208; Cohen’s *d* = 0.48) and the presence of carotid artery plaques (*p* = 0.005 *, *z*-score: −2.77; Cohen’s *d* = 0.54). However, no statistically significant difference was observed when comparing the PTAs of patients with normal results to those with carotid artery stenosis (*p* = 0.07; *z*-score: −1.806).

Figure 2 illustrates a tendency for correlations between carotid–vertebral Doppler ultrasonography results and both the intensities and frequencies of tinnitus. When comparing tinnitus intensity across the four groups, a statistically significant difference was found (*p* = 0.013 *; *H* = 15.71). Further analysis revealed that the group with intimal hyperplasia had significantly higher tinnitus intensity values (*p* = 0.006 *, *z*-score: 2.73; Cohen’s *d* = 0.6). Similarly, the carotid artery plaques group (*p* = 0.041, *z*-score: 2.03; Cohen’s *d* = 0.58) and the carotid artery stenosis group (*p* = 0.005 *, *z*-score: −2.75; Cohen’s *d* = 0.7) also exhibited higher tinnitus intensities compared to the normal results group. Therefore, a more severe change in the carotid arteries may indicate louder tinnitus. Although a similar trend was observed regarding tinnitus frequencies, the differences were not statistically significant (*p* = 0.401; *H* = 2.93). This suggests that more severe changes in the carotid arteries may be associated with louder tinnitus.

**Figure 3:**
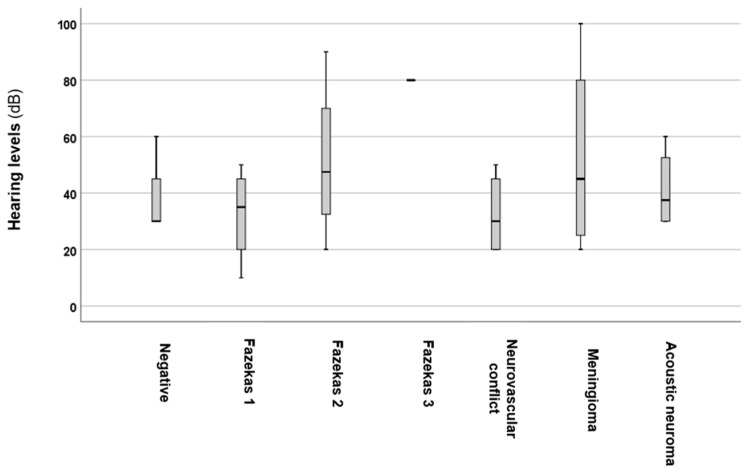
PTA based on alterations detected on brain MRI. The black line that separates the boxes marks the median values. The statistical differences were analysed using the Mann–Whitney *U* test and Kruskal–Wallis test (*p* < 0.05). dB = decibel; PTA = pure-tone average.

**Figure 4:**
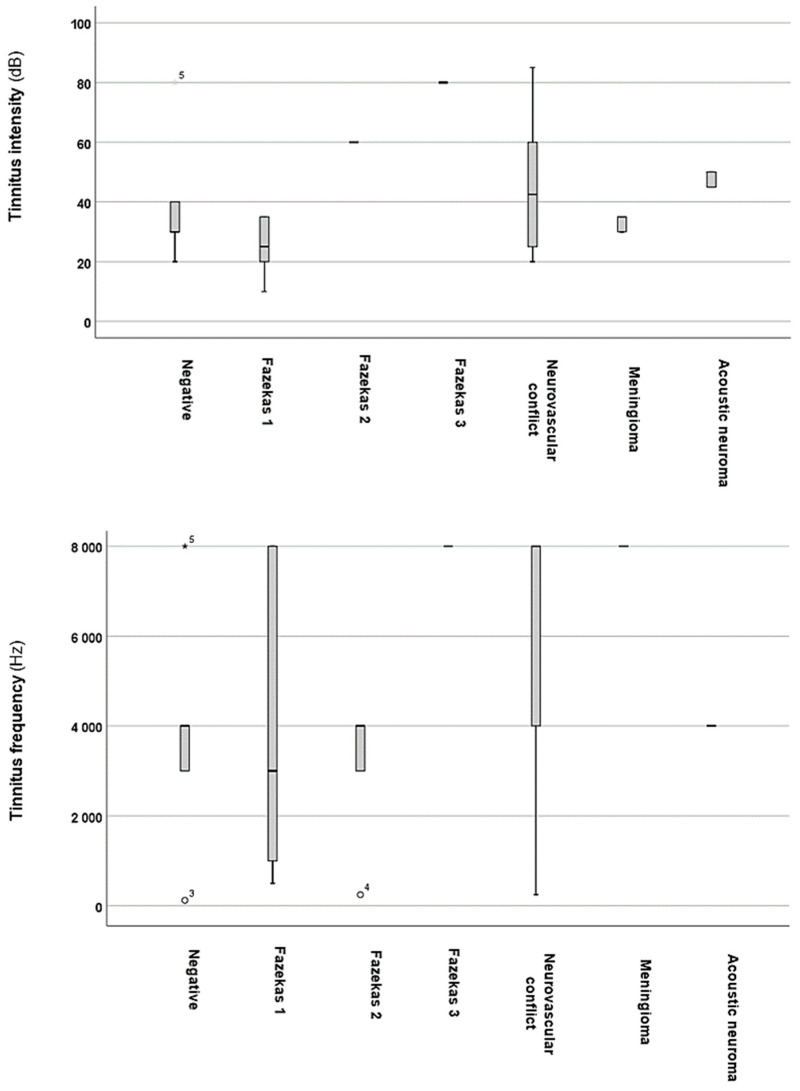
Tinnitus intensities and frequencies based on alterations detected on brain MRI. The black line that separates the boxes marks the median values. The statistical differences were analysed using the Mann–Whitney *U* test and Kruskal–Wallis test (*p* < 0.05 *). dB = decibel; Hz = Hertz. The asterisks/circles and numbers in the figure represent the outliers.

To further investigate the potential effects of brain MRI, changes on PTAs (Figure 3), as well as tinnitus intensity and frequency (Figure 4), were analysed.

Statistical analysis using the Kruskal–Wallis test revealed significant differences (*p* = 0.028 *; *H* = 10.86) among the groups with brain MRI alterations (Figure 3). Further analysis with the Mann–Whitney *U* test indicated significant differences in PTAs for the Fazekas 2 (*p* = 0.02 *, *z*-score: −2.21; Cohen’s *d* = 0.05) and 3 (*p* = 0.0052 *, *z*-score: −3.47; Cohen’s *d* = 0.5) groups when compared to the normal brain MRI group. However, no statistically significant differences were found in the Fazekas 1 (*p* = 0.8; *z*-score: −0.25), neurovascular conflict (*p* = 0.64; *z*-score: −0.45), meningioma (*p* = 0.13; *z*-score: −1.49), or acoustic neuroma (*p* = 0.43; *z*-score: −0.78) groups.

As Figure 4 depicts, the groups with brain MRI alterations exhibit higher tinnitus intensity values compared to those with normal results. Statistical analysis indicates a significant difference (*p* = 0.00097 *; *H* = 18.54) between these groups. Further analyses revealed significantly higher tinnitus intensity values in the Fazekas 2 (*p* = 0.0027 *, *z*-score: 2.98; Cohen’s *d* = 0.28) and Fazekas 3 (*p* = 0.0005 *, *z*-score: −3.45; Cohen’s *d* = 0.3) groups, as well as in the acoustic neuroma group (*p* = 0.019 *, *z*-score: −2.33; Cohen’s *d* = 0.48), when compared to the normal brain MRI group. However, no significant differences were found in the Fazekas 1 (*p* = 0.06; *z*-score: −1.81), neurovascular conflict (*p* = 0.15; *z*-score: −1.42), or meningioma (*p* = 0.23; *z*-score: −1.19) groups. Furthermore, tinnitus frequencies were not significantly (*p* = 0.36; *H* = 4.34) influenced by brain MRI abnormalities.

To further analyse the potential predictors of sensorineural hearing loss, as well as the intensity and frequency of tinnitus, a multinomial logistic regression model was applied. The results are presented in Table 2 and Table 3.

The results presented in Table 2 indicate that abnormalities detected through carotid and vertebral ultrasonography (*p* = 0.000 *; OR: 361.596, 95% CI = 165.303–790.982) and brain MRI abnormalities (*p* = 0.000 *; OR: 348.777, 95% CI = 160.589–757.496) are significant predictors of sensorineural hearing loss. In contrast, other factors included in the model, such as sex and chronic symptoms (lasting more than 3 months), were not found to be significant predictors.

The data presented in Table 3 indicate that abnormalities detected through carotid and vertebral ultrasonography are significant predictors of greater tinnitus intensity (*p* = 0.008 *; OR: 0.055, 95% CI = 0.006–0.472). In contrast, other factors analysed in the model, such as chronic symptoms (lasting more than 3 months) and the location of the symptoms, were not found to be significant predictors. In terms of tinnitus frequency, none of the parameters included in the model were identified as significant predictors.

## 4. Discussion

This investigation examined how abnormalities detected through carotid and vertebral Doppler ultrasonography and brain MRI can influence PTA, as well as the intensity and frequency of tinnitus. The results show that individuals with abnormalities identified in carotid−vertebral ultrasonography had significantly higher PTA values compared to those with normal results. Specifically, conditions such as intimal hyperplasia and arterial plaques were associated with notably elevated PTA levels. Interestingly, carotid artery stenosis did not significantly affect PTA. This may be due to the fact that carotid artery stenosis occurred at relatively lower rates compared to other types of abnormalities. A similar trend was observed regarding tinnitus intensity; however, tinnitus frequency was not significantly affected by the conditions noted in the carotid−vertebral ultrasonography. Additionally, the presence of abnormalities on brain MRI, specifically Fazekas scores of 2 and 3, had a significant impact on PTA. The findings related to the non-significant effects of pathologies observed through carotid and vertebral ultrasonography, as well as brain MRI, are perplexing and require further investigation. One possible explanation for the connection between these alterations and the intensity of tinnitus is that tinnitus intensity may be more closely linked to the severity of hearing loss due to vascular factors. However, the onset of tinnitus frequency could be more complex, potentially influenced by factors such as tonotopic reorganisation, spontaneous neural activity, and neural synchrony [20]. Moreover, the vascular microangiopathy scores and the presence of acoustic neuromas in the cerebellopontine angle regions significantly impacted the intensity of tinnitus, although they did not affect its frequency.

Research on carotid and vertebral sonography concerning sensorineural hearing loss and tinnitus is somewhat limited. One specific study found that individuals with sensorineural hearing loss had significantly higher intima–media thicknesses in both the common carotid artery and the internal carotid artery. However, this study did not show significant increases in peak systolic velocities, vascular diameters, or blood flow related to hearing loss [9]. Another investigation revealed that individuals with carotid artery stenosis exhibited significantly poorer hearing levels in the left ear. Furthermore, analysis using brainstem auditory evoked potentials (BAEPs) showed that the carotid artery stenosis group had significantly increased latencies for waves I, III, and V, as well as extended intervals for I–III, I–V, and III–V waves in both ears [10]. An increased risk of sudden sensorineural hearing loss has also been associated with vertebrobasilar insufficiency [21]. Additionally, a prior study indicated that increased pulsatility and resistance indexes in the internal carotid artery and common carotid artery were linked to a poorer prognosis for hearing loss [22].

In a previous investigation into pulsatile tinnitus, researchers found that patients exhibited significantly elevated end-diastolic velocity and increased intima–media thickness in the internal carotid artery. These findings suggest that tinnitus may be an initial warning sign of serious, potentially life-threatening conditions [7]. Another study reported significantly higher mean flow velocities in the vertebral arteries of patients with tinnitus [8]. Other studies primarily examined the relationship between carotid–vertebral ultrasonography results and the occurrence of pulsatile or objective tinnitus [23,24].

A study examining brain MRI results in patients with unilateral non-pulsatile tinnitus who did not have asymmetrical hearing found that acoustic neuroma (also known as vestibular schwannoma) was detected at a low rate of only 0.3%. Among the 566 scans analysed, there were 134 incidental findings. These findings included conditions such as small vessel disease, cerebellar infarction, arachnoid cysts, empty sella syndrome, and pituitary cysts, among others [25]. However, the findings from the current investigation suggest that these incidental findings may influence the intensity of tinnitus and affect PTA. In a separate investigation, abnormalities related to tinnitus observed on MRIs related to tinnitus were noted in only 2.2% of cases, which increased to 3.2% among patients with unilateral tinnitus. Incidental findings were detected in 41% of participants, and the presence of AICA loops showed no correlation with abnormalities in ABRs [26]. In the current investigation, no relationship was found between neurovascular conflict and the severity of sensorineural hearing loss, nor between neurovascular conflict and the intensities and frequencies of tinnitus. However, MRI and MRA have proven effective in identifying the causes of pulsatile tinnitus [27]. One investigation found that patients with unilateral non-pulsatile tinnitus and asymmetrical sensorineural hearing loss are more likely to show abnormalities on brain MRI [28]. In cases of sensorineural hearing loss accompanied by MRI findings, there was a higher ratio of relevant contrast-enhanced brain MRI results alongside abnormal ABR findings. Tinnitus was more pronounced in patients with brain MRI pathologies [29]. These findings support the results of the current study. Moreover, age-related sensorineural hearing loss has been linked to increased brain atrophy and reduced white matter integrity [30].

While this investigation has notable strengths, several limitations need to be addressed. First, there was no specific control group of individuals without sensorineural hearing loss and tinnitus. This absence makes it difficult to determine whether the vascular and MRI abnormalities observed were genuinely associated with the audiological findings or if they were merely incidental. Moreover, the potential effects of other factors, such as hypertension, diabetes mellitus, medication side effects, or noise exposure, could not be analysed, which may introduce further bias. Additionally, the study only examined pathological changes as broad categories, without analysing specific parameters, such as those derived from carotid–vertebral ultrasonography. The duration of tinnitus varied widely among participants, which could introduce bias. Nevertheless, given the inclusion of a significant number of tinnitus patients, we believe these findings provide valuable insights into the relationship between tinnitus, sensorineural hearing loss, and medical imaging results. Further prospective studies that consider these factors are necessary.

## 5. Conclusions

This study identified a significant association between elevated PTA levels and abnormalities found in carotid and vertebral ultrasonography, particularly regarding intima hyperplasia and the presence of carotid artery plaques. Furthermore, these ultrasonographic abnormalities were significantly related to the intensity of tinnitus, though they did not influence the frequencies of tinnitus. Among brain MRI findings, higher grades of microvascular lesions were shown to impact PTA, while tinnitus intensity was also influenced by the presence of acoustic neuroma. However, tinnitus frequencies were not significantly affected. These results underscore the importance of conducting carotid–vertebral ultrasonography and brain MRI examinations in clinical practice, as they may provide targeted options for the treatment of tinnitus and sensorineural hearing loss. The findings from recent investigations emphasise the significance of vascular risk factors in relation to sensorineural hearing loss and the intensity of tinnitus. Consequently, it is essential to consider these factors in everyday practice when managing tinnitus and sensorineural hearing loss. Since these vascular factors can influence audiological outcomes, patients should be referred for further assessments to identify potential underlying conditions that may be contributing to these issues through medical imaging.

## Figures and Tables

**Table 1:** Basic demographic and clinical data of the study population (*n* = 423). dB = decibel; Hz = Hertz; IQR = interquartile range; PTA = pure-tone average; Q1 = first quartile; Q3 = third quartile.

Category	Values
Age (median years; IQR, Q1–Q3)	51 (24; 41–65)
Sex (men/women)	184/239
Tinnitus onset (median months; IQR, Q1–Q3)	12 (33; 3–9)
Tinnitus location right, *n* (%) left, *n* (%) bilateral, *n* (%)	103 (24.3%) 134 (31.7%) 186 (44%)
PTA (median dB; IQR, Q1–Q3)	30 (25; 20–45)
Tinnitus intensity (median dB; IQR, Q1–Q3)	30 (27.5; 20–47.5)
Tinnitus frequency (median Hz; IQR, Q1–Q3)	4000 (6000; 2000–8000)

**Table 2:** A multinomial logistic regression model to analyse the predictors for sensorineural hearing loss. CI = confidence interval, OR = Odds ratio, Std. = standard. The significant results (*p* < 0.05) are indicated with an asterisk (*).

Dependent	Predictor	*β*	Std. Error	*p*-Value	OR	95% CI (Lower Bound)	95% CI (Upper Bound)
Sensorineural hearing loss	Carotid–vertebral ultrasonography alteration	5.891	0.399	0.000 *	361.596	165.303	790.982
	Brain MRI alteration	5.854	0.396	0.000 *	348.777	160.589	757.496
	Sex	−0.262	0.330	0.428	0.770	0.403	1.471
	Chronic symptoms	−0.533	0.368	0.148	0.587	0.285	1.208

**Table 3:** A multinomial logistic regression model to analyse the predictors of tinnitus intensity and frequency. CI = confidence interval, OR = Odds ratio, Std. = standard. The significance level was established at *p* < 0.05. The significant results (*p* < 0.05) are indicated with an asterisk (*).

Dependent	Predictor	*β*	Std. Error	*p*-Value	OR	95% CI (Lower Bound)	95% CI (Upper Bound)
Tinnitus intensity (higher intensity = over 30 dB)	Carotid–vertebral ultrasonography alteration	−2.896	1.094	0.008 *	0.055	0.006	0.472
	Brain MRI alteration	0.197	0.601	0.744	1.217	0.375	3.955
	Sex	−0.199	0.552	0.718	0.819	0.278	2.418
	Chronic symptoms	−0.604	0.619	0.330	0.547	0.162	1.841
	Right-sided tinnitus	−0.603	0.649	0.352	0.547	0.153	1.950
	Left-sided tinnitus	−1.231	0.723	0.089	0.292	0.071	1.205
	Bilateral tinnitus	0.230	0.328	0.000 *	1.259	0.662	2.395
Tinnitus frequency (higher frequency = over 4000 Hz)	Carotid–vertebral ultrasonography alteration	0.336	0.488	0.491	1.400	0.538	3.642
	Brain MRI alteration	0.100	0.447	0.824	1.105	0.460	2.655
	Sex	−0.074	0.423	0.861	0.929	0.406	2.126
	Chronic symptoms	0.075	0.509	0.883	1.078	0.398	2.921
	Right-sided tinnitus	−0.021	0.578	0.971	0.979	0.315	3.039
	Left-sided tinnitus	0.615	0.367	0.094	1.849	0.900	3.799
	Bilateral tinnitus	−0.634	0.496	0.201	0.530	0.201	1.402

## Data Availability

The data presented in this study are available on request from the corresponding author due to reasonable request.

## Abbreviations

The following abbreviations are used in this manuscript:

AICA	anterior inferior cerebellar artery
ABR	auditory brainstem response
BAEP	brainstem auditory evoked potential
dB	decibel
DWI	diffusion-weighted imaging
FLAIR	fluid-attenuated inversion recovery
Hz	Hertz
IBM	International Business Machines Corporation
IQR	interquartile range
MRA	magnetic resonance angiography
MRI	magnetic resonance imaging
PTA	pure-tone average
SPSS	Statistical Package for the Social Sciences
Q1	first quartile
Q3	third quartile
SWI	susceptibility-weighted imaging
